# Purification/annealing of graphene with 100-MeV Ag ion irradiation

**DOI:** 10.1186/1556-276X-9-126

**Published:** 2014-03-17

**Authors:** Sunil Kumar, Ambuj Tripathi, Fouran Singh, Saif Ahmad Khan, Vikas Baranwal, Devesh Kumar Avasthi

**Affiliations:** 1 Materials Science Group, Inter University Accelerator Centre, Aruna Asaf Ali Marg, New Delhi 110067, India; 2 Nanotechnology Application Centre, University of Allahabad, Allahabad 211002, India

**Keywords:** Graphene, Ion irradiation, Annealing, Disorder parameter, Inelastic thermal spike model

## Abstract

Studies on interaction of graphene with radiation are important because of nanolithographic processes in graphene-based electronic devices and for space applications. Since the electronic properties of graphene are highly sensitive to the defects and number of layers in graphene sample, it is desirable to develop tools to engineer these two parameters. We report swift heavy ion (SHI) irradiation-induced annealing and purification effects in graphene films, similar to that observed in our studies on fullerenes and carbon nanotubes (CNTs). Raman studies after irradiation with 100-MeV Ag ions (fluences from 3 × 10^10^ to 1 × 10^14^ ions/cm^2^) show that the disorder parameter *α*, defined by *I*_D_/*I*_G_ ratio, decreases at lower fluences but increases at higher fluences beyond 1 × 10^12^ ions/cm^2^. This indicates that SHI induces annealing effects at lower fluences. We also observe that the number of graphene layers is reduced at fluences higher than 1 × 10^13^ ions/cm^2^. Using inelastic thermal spike model calculations, we estimate a radius of 2.6 nm for ion track core surrounded by a halo extending up to 11.6 nm. The transient temperature above the melting point in the track core results in damage, whereas lower temperature in the track halo is responsible for annealing. The results suggest that SHI irradiation fluence may be used as one of the tools for defect annealing and manipulation of the number of graphene layers.

**PACS:**

60.80.x; 81.05.ue

## Background

Graphene is a new member of the carbon family that has two-dimensional honeycomb lattice structure [1,2]. It is a basic building block for other carbon materials of different dimensionalities. It can be wrapped into zero-dimensional fullerenes, rolled up into one-dimensional carbon nanotube and stacked together to form three-dimensional graphite [3]. The high mechanical stability together with ballistic electron transport properties makes graphene a strong replacement for silicon-based semiconductor technology. At present, electron beam lithography (EBL) is widely used to fabricate nanoelectronic devices. For example, EBL is often adapted for fabrication of drain source and gate electrodes in graphene field-effect transistor (GFET) structure, while graphene channel is fabricated by alternate means. Similarly, the use of scanning electron microscope (SEM) for observation and testing of GFET structures is very common [4]. Electron beam itself has also been used for the formation of epitaxial graphene on the surface of a 6H-SiC substrate by irradiation [5]. Hence, in conventional EBL, the electron beam irradiation of graphene and consequent damage become unavoidable. For this reason, the electron beam irradiation-induced modifications in graphene [4,6-11], especially in the energy regime relevant for lithography and microscopy, are widely being studied.

The graphene properties are sensitive to defects. For example, it has been reported that metal to insulator transition occurs with small introduction of defects in graphene perfect lattice [12]. Hence, for the proper use of graphene-based devices, the studies on defect control and behaviour of graphene in defect environment are very important. Low-energy (keV to few MeV) ion beam irradiation is one of the important tools to engineer defects in a controlled manner. Though the effect of ion irradiation and collision cascades in solid have been known for a long time [13], there are only few studies on the interaction of ions with two-dimensional (2D) crystals such as graphene [14] and how the irradiation-induced defects can be used to engineer their electronic properties [15]. It has been theoretically shown that vacancies can induce 2D magnetic ordering in graphene [16] besides modifying the electronic states near the Fermi level originating from the shape of electronic bands [17-19] near the Dirac point [20,21]. Ripple formation induced by 1-keV Ar ion irradiation, showing a possibility of tailoring of few-layer graphene electronic band structure with inter-layer coupling, has been demonstrated [22]. Irradiation study of graphene with 90-eV Ar^+^ ions has shown that the disorder produced in graphene leads to two competing processes resulting in an initial increase in the formation of defects as depicted by the increase in *I*_D_/*I*_G_ ratio followed by its decrease at higher fluence [23]. The defect studies with 500-keV C^+^ ions [24] and 2-MeV protons [25] show that a single-layer graphene is more sensitive to irradiation damage than a multilayer graphene, and its damage follows the behaviour proposed by Tuinstra and Koenig for nanographite [26]. Structural transformation from nanocrystalline graphene to amorphous carbon and corresponding electrical transition from Boltzmann diffusion to carrier hopping transport induced by 30-keV Ga ions have been demonstrated by Zhou et al. [27]. Graphene is also supposed to be mechanically stable under irradiation. Atomistic simulations have demonstrated that irradiated graphene, even with a high vacancy concentration, does not exhibit any sign of instability, thus showing its applicability as robust windows [28]. Zhao et al. studied the defect production in graphene, induced directly by incident ion and also indirectly by backscattered and sputtered particle with the help of molecular dynamics method [29]. Yang et al. proposed the thinning of graphene layer by the use of nitrogen plasma irradiation and post-annealing treatments [30].

As mentioned above, most of the irradiation studies on graphene are focused on damage by electron and low-energy ions, and there are only few reports available on the ion irradiation studies of graphene at high energy [31]. The study of high-energy ion irradiations, such as mega-electron volt protons, is also important due to the potential use of graphene devices in space applications, specially solar cells [32]. Though the irradiation-induced damage is very common, it has been demonstrated that swift heavy ions (SHI), with carefully selected ranges of fluence, can also be used for annealing of defects in graphene. We have earlier observed the annealing/ordering effect in fullerenes and multiwalled carbon nanotubes (MWCNT) at lower fluences after irradiation with 200-MeV Au, 60-MeV Ni and 55-MeV C ions [33,34].

## Methods

In the present work, graphene flakes grown with chemical vapour deposition (CVD) technique on Si/SiO_2_/Ni substrate from Graphene Laboratories Inc., USA were used. Though mechanically cleaved graphene samples generally provide better quality single-layer graphene, we have used commercially available CVD-grown samples which are available in larger size, with industrial applications in mind. Irradiation is carried out with 100-MeV Ag^7+^ ions using 15-MV Pelletron accelerator at IUAC, New Delhi, India, at fluences of 3 × 10^10^, 1 × 10^11^, 3 × 10^11^, 1 × 10^12^, 3 × 10^12^, 1 × 10^13^, 3 × 10^13^ and 1 × 10^14^ ions/cm^2^. The pressure in the irradiation chamber was of the order of 10^−6^ mbar, and the average ion current was 1 particle nanoampere (pnA; 1 pnA = 6.25 × 10^9^ ions/s). The sample was cut into pieces of 1 × 1 cm^2^ size for irradiation. Half of the area of each sample was masked with thin aluminium foil during irradiation to keep half the portion unirradiated for comparing the results.

The surface topography was studied using Digital Instruments Nanoscope IIIa atomic force microscope (AFM; Santa Barbara, CA, USA) in the tapping mode. Raman measurements were carried out using Renishaw inVia microRaman set-up (Gloucestershire, UK). Initially, we performed Raman measurements on pristine sample at several spots with different laser powers to check the self-heating effect. We took 10% (2 mW) of the laser power, which is below the threshold value for laser-induced heating, for measurement on all the samples. Therefore, self-annealing effect during Raman measurement is ruled out.

Raman spectra of pristine (unirradiated) as well as irradiated portions of each sample were recorded at four different spots (flakes) using Ar ion laser with excitation wavelength of 514.5 nm and averaged out for further analysis. The size of the laser spot is approximately 2 μm with × 50 optical magnification of the attached optical microscope. The peak intensities have been obtained by fitting the peaks assuming Lorentzian distribution.

## Results and discussion

**Figure 1:**
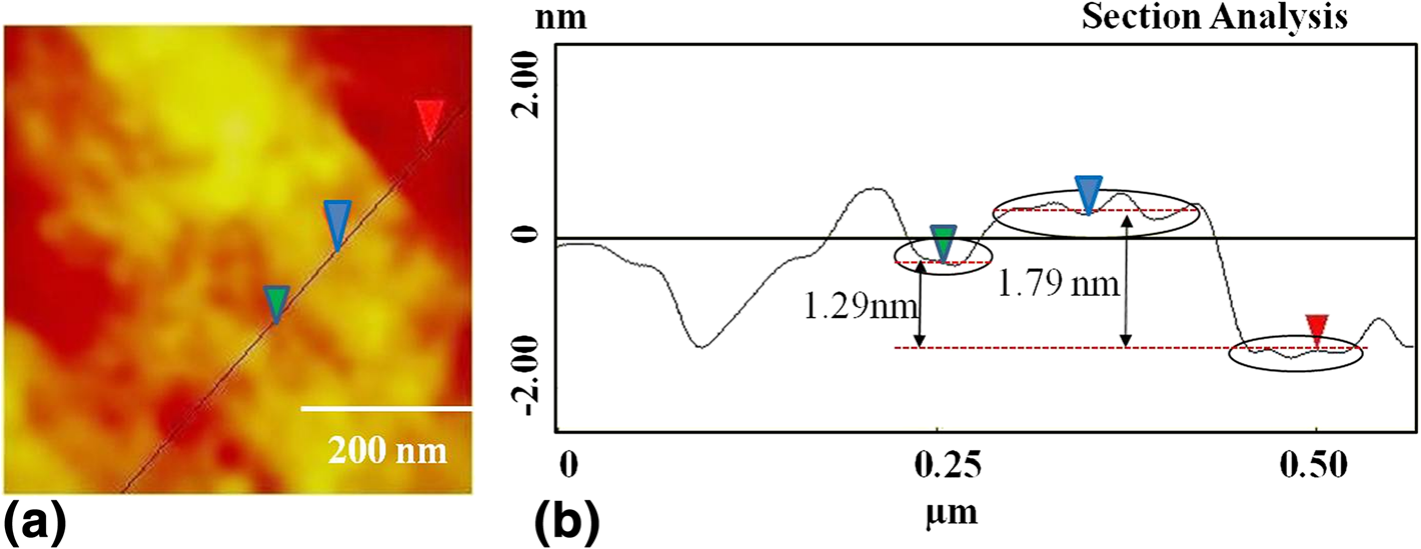
Typical AFM topographic image of the unirradiated sample near the edge (a) with its line profile (b).

A typical AFM topographic image of the sample is shown in Figure 1a. The step height analysis at different parts of the sample shows that the sample consists of mostly three to four graphene layers. The average size of the graphene flakes is 3 to 4 μm. The line profile of the sample near the edge of the flakes (Figure 1b) shows a film height of 1.79 and 1.29 nm which corresponds to the four- and the three-layer thickness, respectively, and is in agreement with the sample specification.

The film thickness shows a linear relation of *h* ~ 0.35*n* + *t*_0_, where *h* is the measured height, *t*_0_ is the approximate distance between the first graphene layer and the substrate and *n* is the number of layers. Substrate to the first layer height *t*_0_ is approximately 0.6 to 0.7 nm [35].

**Figure 2:**
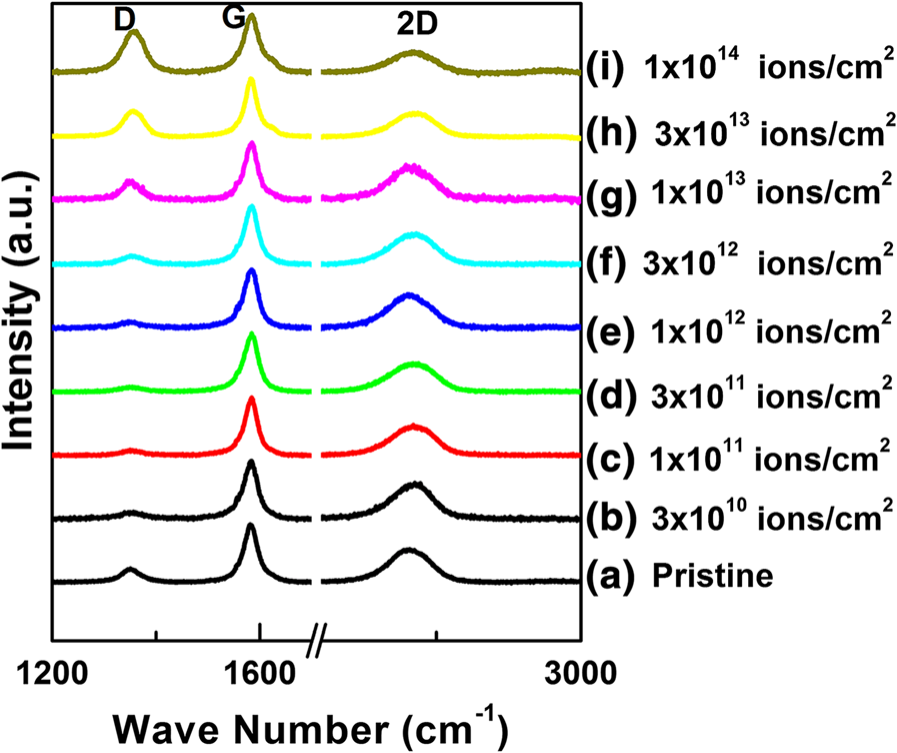
**Raman spectra of graphene before and after irradiation with 100-MeV Ag ions at different fluences (in ions/cm**^**2**^**).**

The evolution of Raman spectra of pristine as well as irradiated part is shown in Figure 2. Raman spectra of graphene consist of three main peaks: D peak (centred around 1,350 cm^−1^), G peak (centred around 1,580 cm^−1^) and 2D peak (centred around 2,700 cm^−1^). The G peak is the characteristic peak of graphitic material, and its origin is due to *E*_2*g*_ symmetry mode near the *k* = 0 point in the first Brillouin zone. It is the first-order process and a stretching vibrational mode of the carbon atom both in the rings and in the chain [36]. Any change in this vibration affects the intensity, full width at half maxima (FWHM) and position of the peak. D and 2D peaks are the second-order processes and involve double resonance phenomena. D peak requires defects in the perfect lattice for activation and appears due to *A*_1*g*_ symmetry phonons near the *K*-zone boundary. In the absence of defect, these phonons are Raman inactive due to the momentum conservation in the scattering [37]. They become active in the presence of structural disorder as described by the double resonance model [37]. So, the intensity of D peak is directly related to the disorder or defect present in graphene. 2D peak is the second order of D peak and arises due to double resonance and involves complex transitions explained in detail by Ferrari et al. [37].

The Raman spectra of unirradiated and irradiated graphene at different fluences with 100-MeV Ag ions are shown in Figure 2. The spectra have been normalized to keep the height of G peak the same. The spectra have also been staggered in the *y*-axis for clarity. It has been observed that D peak areal intensity first decreases up to a fluence of 3 × 10^11^ ions/cm^2^ and then starts to increase with increasing fluences up to 1 × 10^14^ ions/cm^2^.

**Figure 3:**
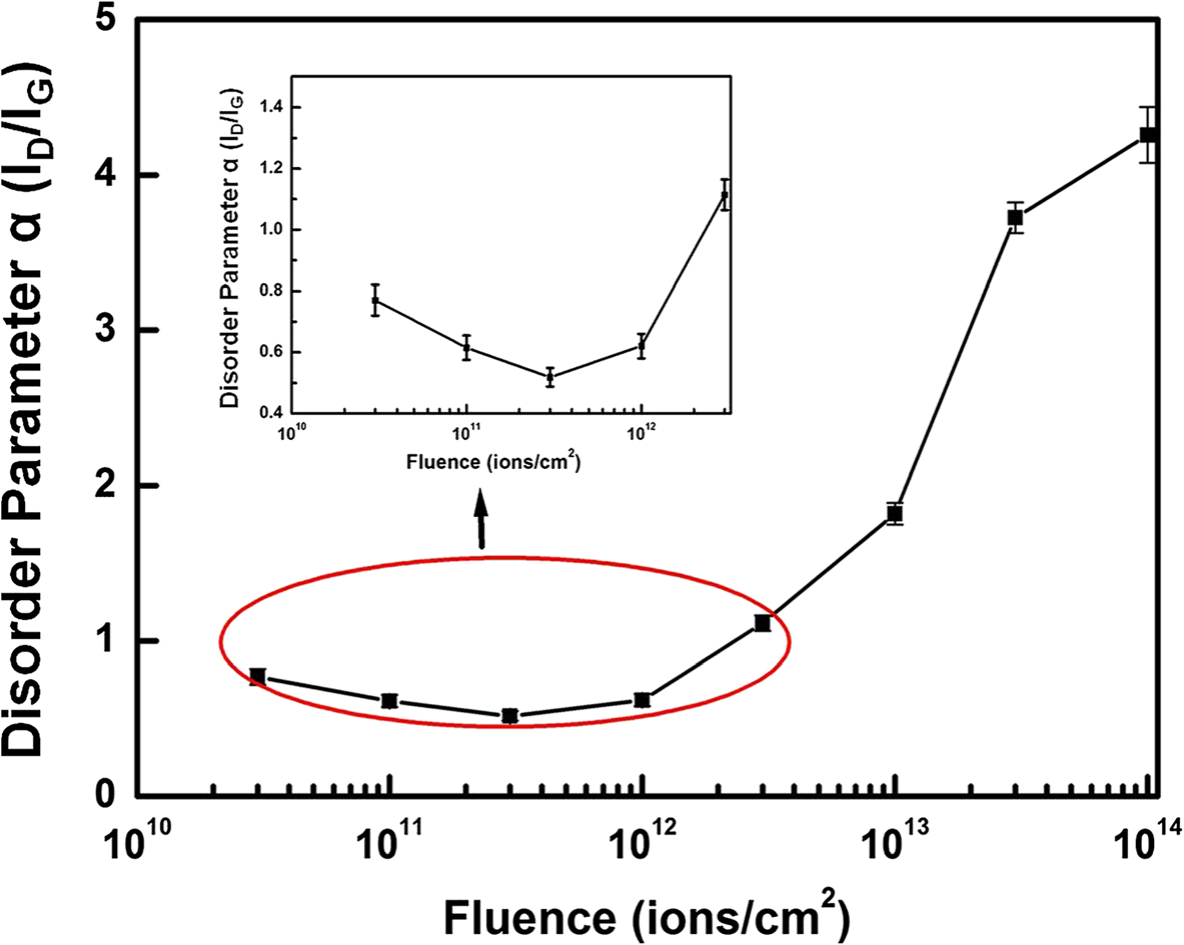
**The plot of disorder parameter *α *** **(*I***_**D**_**/*I***_**G**_**) with fluence.**

The defects induced in carbon-based materials are studied using disorder parameter *α* defined by *I*_D_/*I*_G_ ratio, where the *I*_D_ and *I*_G_ are the intensity of D and G peaks, respectively, in the Raman spectrum. It measures the disorder present in the sample which may be due to defects or misalignment in the graphene sample. Samples of lower disorder have low value of *α*, and a high value of *α* indicates larger amount of disorder. Since there are variations in different parts of the sample, we have normalized the value of *I*_D_/*I*_G_ of irradiated part with the value of *I*_D_/*I*_G_ of pristine part, and these normalized ratios have been studied for further analysis. The variation of *α* for different fluences is plotted in Figure 3.

It is clear from Figure 3 that a small decrement in the value of *α* is observed up to the fluence of 3 × 10^11^ ions/cm^2^, and with further increase in the fluence, there is an increase in its value. This initial decrease signifies ordering or purification of graphene at low fluences and is induced by energy dissipated by the incident energetic ion. To the best of our knowledge, this is the first kind of report on the ordering or purification of graphene. Electron beam irradiation studies [9,38-40] have only shown increase in defects, whereas we have observed decrease in defects at lower fluences. The data with electron beam irradiation is available at a higher fluence only (more than 600 s). It is possible that measurements at a lower fluence with electron beam irradiation (say approximately 10 s) may show annealing. We have also irradiated samples for 10 s at the lowest fluence.

**Figure 4:**
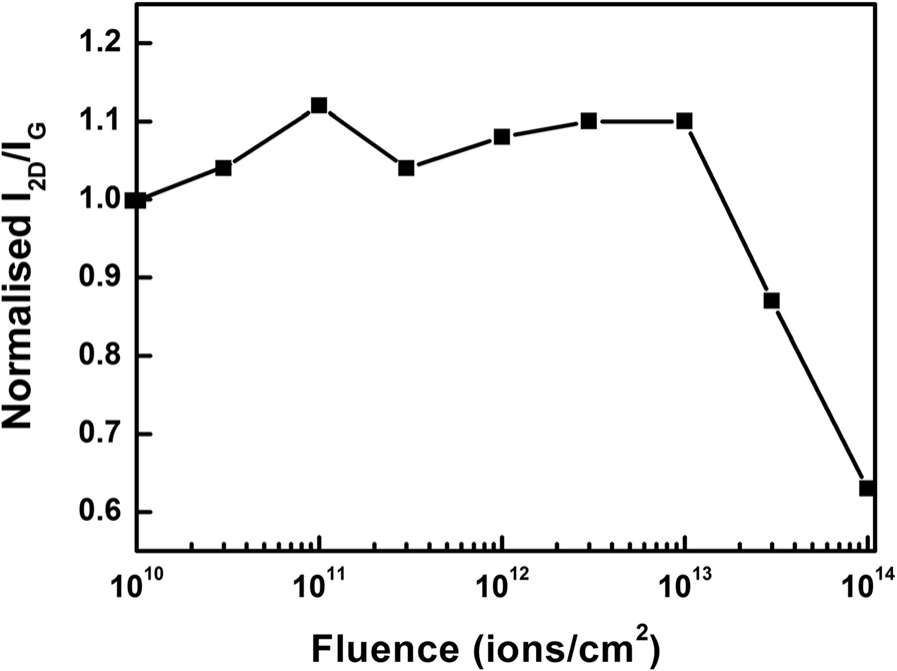
**The plot of *I***_**2D**_**/*I***_**G **_**with fluence.** The values of *I*_2D_/*I*_G_ are normalized to the value of *I*_2D_/*I*_G_ for the unirradiated sample.

The small increment in intensity ratio *I*_2D_/*I*_G_ at a lower fluence also confirms the crystal quality improvement as shown in Figure 4. At higher fluences above 1 × 10^13^ ions/cm^2^, the ratio *I*_2D_/*I*_G_ decreases sharply due to higher defect density.

**Figure 5:**
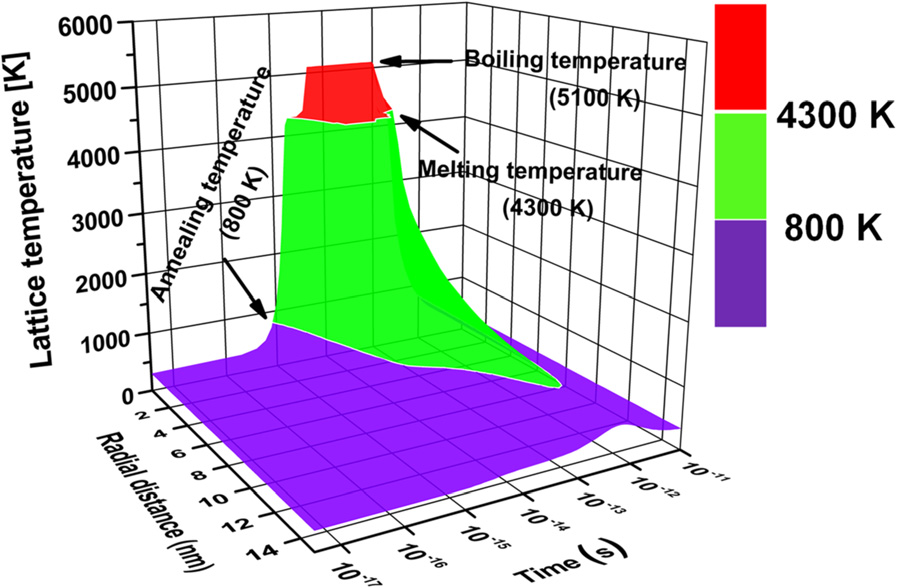
Simulated three-dimensional plot of lattice temperature evolution.

In the present case, as the energetic ion passes through the film, a large fraction of its energy is transferred to the electronic subsystem of the target material by inelastic collisions. According to the thermal spike model, this results in high transient local energy density along the ion path. The deposited energy is transferred to the atomic system by electron-phonon coupling which generates a localized heating of the atomic system in the ion track of nanometric dimension. This results in the transient thermal spike lasting for a few picoseconds. Moreover, the temperature shows a gradual radial decay as the radial distance from the ion track increases [41]. The spatial and temporal evolution of temperature can be simulated using inelastic thermal spike (i-TS) code [42]. Since the values of all the parameters used in the simulation are not available for graphene, we have used the values for crystalline graphite which has similar sp^2^-bonded structure [42]. The temperature thus calculated will give only an approximate value for graphene. Since the graphene sample used in this experiment mostly contains three to four layers, thermal conductivity is less than for single/bi-layer graphene, but comparable to highly oriented pyrolytic graphite (HOPG) [43]. Hence, our simulated temperature distribution is also expected to be closer to HOPG which would have been different in the case of single/bi-layer graphene samples. Moreover, these simulated temperature profiles for crystalline graphite HOPG will also give us an upper limit for the core and halo radii discussed in the next section, as the larger thermal conductivity and mean free path for graphene is expected to result in a lower value of electron-phonon coupling constant *g* as compared to graphite (*g* = 3 × 10^13^ W/cm^3^/K) and subsequently result in a larger calculated value of core and halo radius. Figure 5 shows the simulated three-dimensional (3D) plot of lattice temperature evolution with time at different radial distances from the center after 100-MeV Ag ion impact.

**Figure 6:**
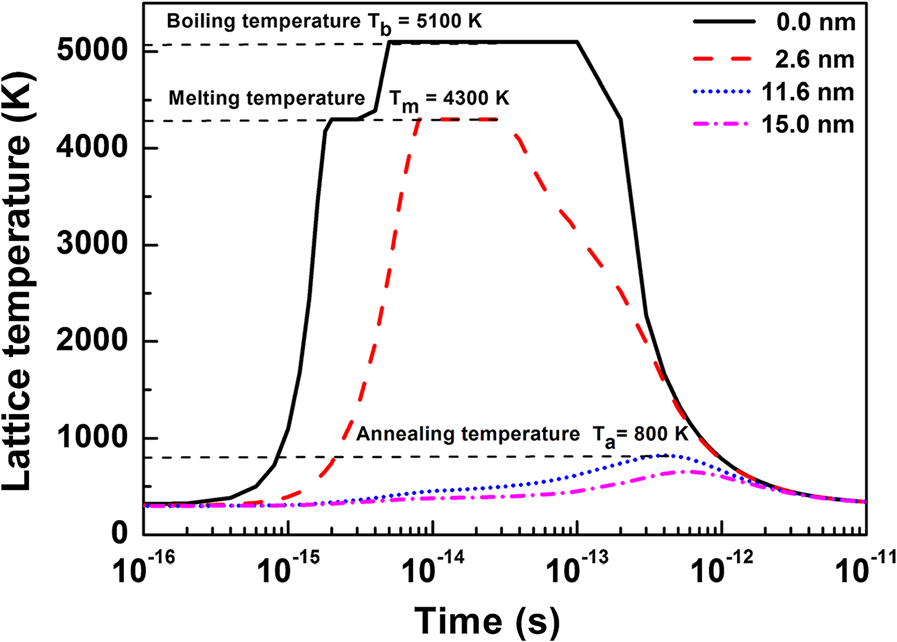
Temporal distribution of lattice temperature.

The temporal distribution of lattice temperature as projected from the 3D plot for radial distances of 0.0 nm (centre of the track core), 2.6 nm, 11.6 nm and 15.0 nm are plotted in Figure 6. As we move away from the core centre, with increasing radial distance, the maximum temperature reached is lower than that reached at the core. The radii of 2.6 and 11.6 nm for the plot have been chosen in such a way that the temperatures reached in lattice are sufficient for melting (4,300 K) and annealing (800 K), respectively, for these two radii.

**Figure 7:**
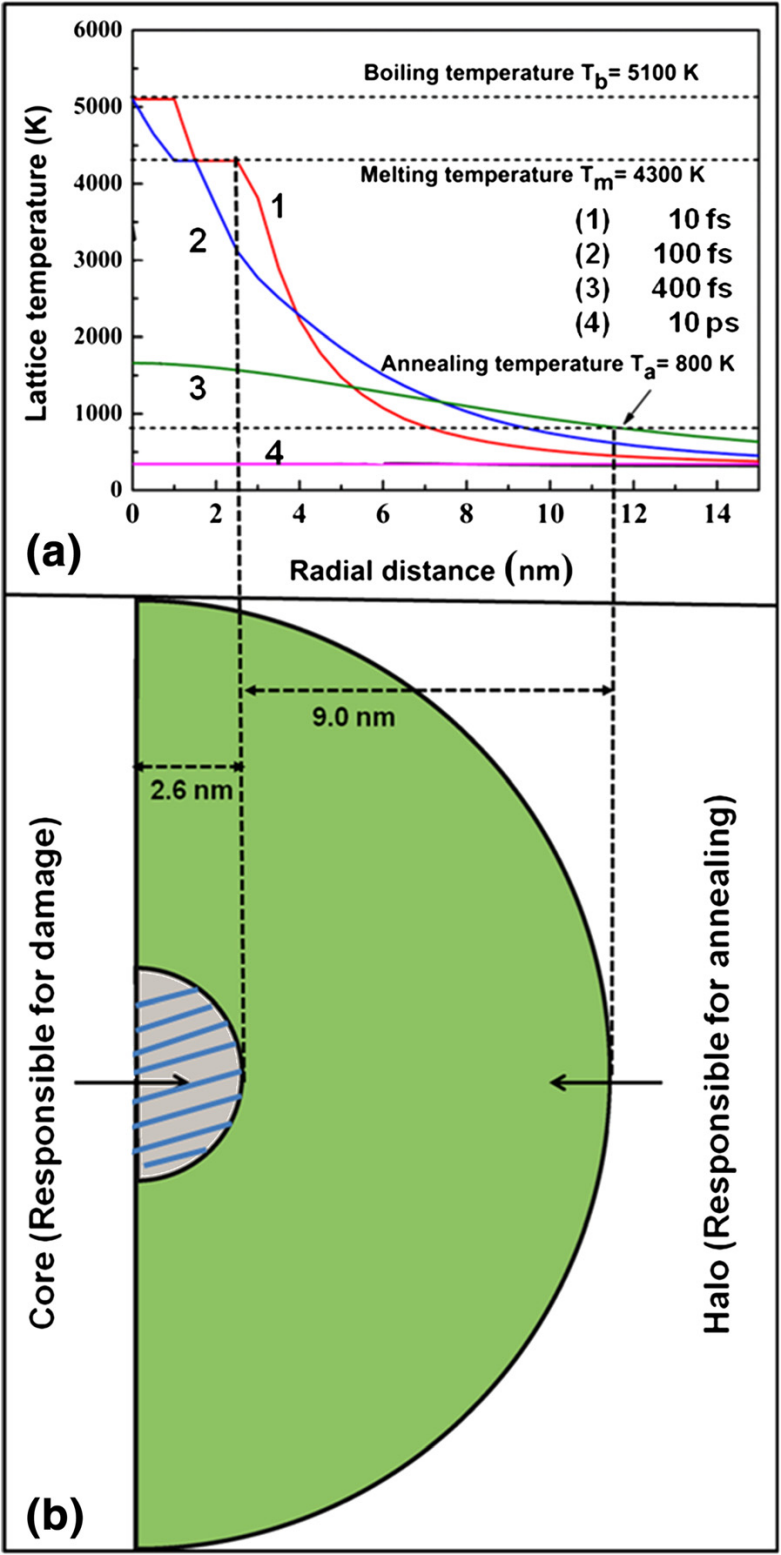
Radial distribution of lattice temperature (a) and pictorial representation of core and halo regions of an ion track (b).

The temperature higher than the melting temperature is reached in a radius of 2.6 nm, thus creating a damaged molten zone along the ion trajectory, referred as the track core. Similarly, temperatures above 800 K, essential for graphene annealing [44], are reached in an annular region of radii from 2.6 to 11.6 nm surrounding the core (referred as the track halo). The radial distribution of lattice temperature as projected from the 3D plot is shown in Figure 7a. The radial temperature profile is plotted for 10 fs, 100 fs, 400 fs and 10 ps after the ion impact. From the curve (1), it is clear that within 10 fs a maximum temperature of approximately 5,000 K is reached at the centre of the core. The maximum temperature reached is lower with increasing radial distances (curves (1) and (2)). As discussed above, temperatures above 800 K are reached in the radius up to approximately 11.6 nm, (curve (3)). The pictorial representation of core and halo regions surrounding the ion track is shown in Figure 7b.

The damage and annealing occurs simultaneously. However, because the ratio of the core and halo radii is approximately 1:4 (from Figure 7b), annealing of defect occurs in much larger area rather than irreversible damage of graphene by the core region. Therefore, the damage is insignificant at low-fluence irradiation. At higher fluences, the core region areas start to overlap, and the damaged area increases. The halo regions are not capable to anneal the damage created in the core regions. From these values of radii for the core and halo, fluences when the core and halo regions are expected to start overlapping are obtained as 5.5 × 10^12^ and 3.4 × 10^11^ ions/cm^2^, respectively. Hence, we can expect that annealing will increase up to a fluence of 3.4 × 10^11^ ions/cm^2^ when halos cover the sample. Beyond this fluence, the region damaged by the track core starts to dominate. Kumar et al. have observed similar behaviour in carbon nanotubes due to ion irradiation [33]. The purification of carbon nanotube is previously reported by laser irradiation and heat treatment also [45,46].

To compare the radii of halo and core regions obtained from inelastic thermal spike calculations, we fitted our experimental data as shown in Figure 3 to estimate the core and halo radii. The equation used for fitting is

(1) $$\frac{I_{D}}{I_{G}}=ae^{-\sigma_{1}\;\phi }+b\left(1-e^{-\sigma_{2}\;\phi }\right)$$

**Figure 8:**
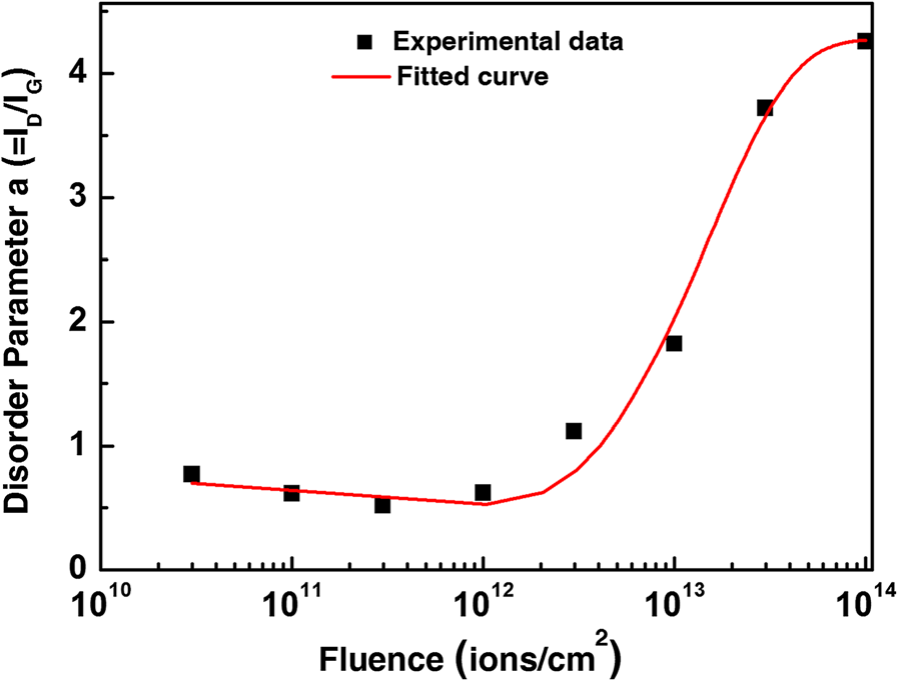
**The simulated curve for disorder parameter *α *** **(*I***_**D**_**/*I***_**G**_**) with fluence.**

In Equation 1, the first term represents exponential decay due to the annealing effect of irradiation in an annular region and the second term represents exponential increase due to damage effects within the ion track core. Constants *a* and *b* are the values of *I*_D_/*I*_G_ when the fluence *ϕ* is zero and infinity, respectively. The values of *σ*_1_ and *σ*_2_ give cross sections for annealing and damage, respectively. For the best fit, the values of parameters *a*, *σ*_1_, *b* and *σ*_2_ were obtained as 0.71 ± 0.1, (1.0 ± 0.1) × 10^−12^ cm^2^, 4.3 ± 0.2 and (6.7 ± 0.8) × 10^−14^ cm^2^ respectively, with chi-square value of 0.04. The simulated curve with the above parameters is shown in Figure 8.

*σ*_1_ and *σ*_2_, respectively, represent the cross-sectional area of cylindrical regions which are annealed and damaged by an incident ion. The annular halo and core radii estimated from the value of *σ*_1_ and *σ*_2_ are 5.7 and 1.4 nm, respectively. The overlapping fluence calculated with this core radius is 1.6 × 10^13^ ions/cm^2^. The halo and core radii observed for graphene are found to be smaller in comparison to radii 11.6 and 2.6 nm obtained from thermal spike calculations. The smaller core and halo radii can be explained in terms of difference in the strength of electron-phonon coupling (*g* value) of graphene and graphite. Larger thermal conductivity and mean free path may lead to a lower value of *g* in the case of graphene as compared to graphite (*g* = 3 × 10^13^ W/cm^3^/K) and subsequent estimated lower value for core and halo radii.

**Figure 9:**
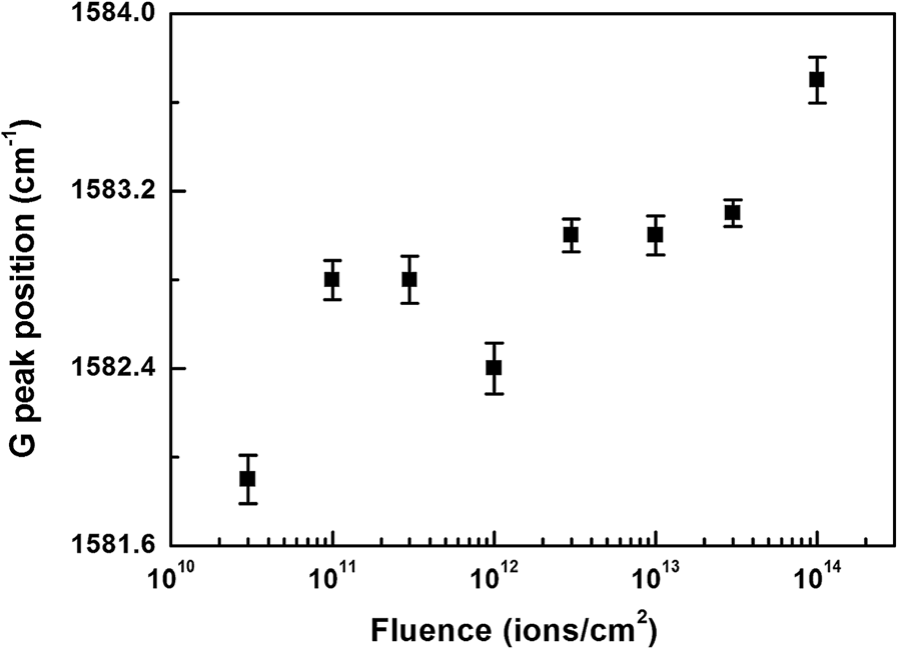
Plot of shift in G peak position with fluence.

We have also observed that G peak position shows a shift towards higher wave number from 1,581.9 to 1,583.7 cm^−1^ at fluence of 1 × 10^14^ ions/cm^2^ (Figure 9). The upshift in G peak occurs mainly due to strain induced in the graphene and reduction in layer number. However, the shift due to strain is reported to be much higher approximately 20 cm^−1^[47,48] than the observed value of upshift approximately 1.8 cm^−1^ in our case. Therefore, the small shift in G peak towards higher wave number is interpreted as decrease in the number of layers from three to four to one to two [35,49] with irradiation.

It is expected that the intensity ratio *I*_2D_/*I*_G_ would increase at higher fluence when the number of layers decreases [35,37,50], but at the same time due to higher defect density, the ratio *I*_2D_/*I*_G_ decreases much more rapidly [51]. Therefore, overall effect is decreased in *I*_2D_/*I*_G_ ratio as shown in Figure 4. It is also known from the reported work that the FWHM of 2D peak increases with the increase of defects in graphene [52,53]. Hence, at a higher fluence above 1 × 10^13^ ions/cm^2^, the expected decrease in FWHM of 2D peak due to layer number reduction is not observed.

**Figure 10:**
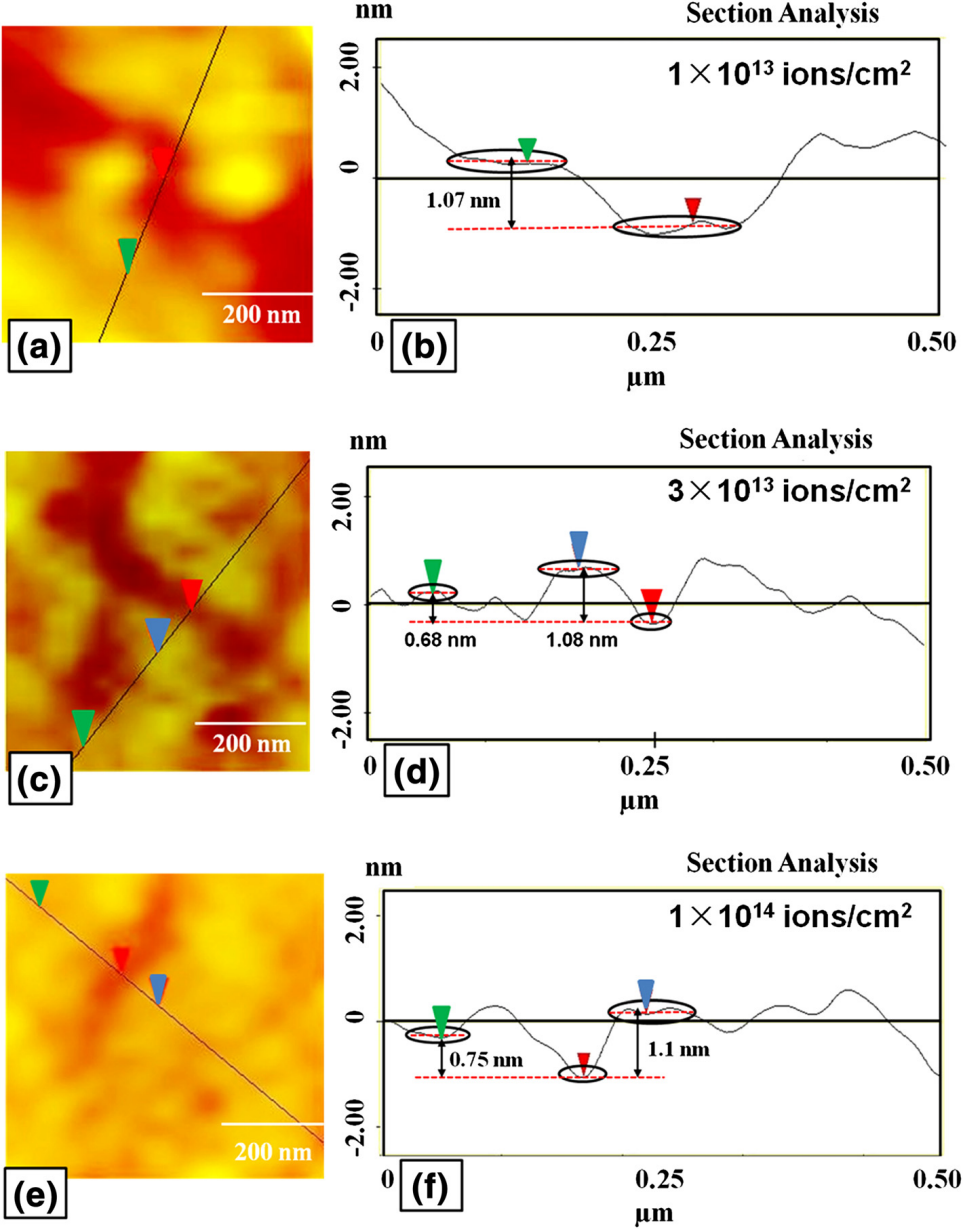
**Atomic force microscopy imaging and corresponding section analyses of irradiated part of samples.** Atomic force microscopy imaging of irradiated part of graphene is shown at fluences **(a)** 1 × 10^13^, **(c)** 3 × 10^13^ and **(e)** 1 × 10^14^ ions/cm^2^, and corresponding section analyses is shown in **(b)**, **(d)** and **(f)**, respectively.

Atomic force microscopy imaging (Figure 10) also confirms the reduction in number of layers at different places of irradiated samples and shows that at higher fluence (above 1 × 10^13^ ions/cm^2^) there is more coverage of one- and two-layer graphene instead of three to four layers observed before irradiation. The ion irradiation-induced ablation in graphene can be used for controlling the number of layers with spatial selectivity. Similar reduction in the number of graphene layers with laser ablation above a given threshold laser energy density has been demonstrated by Dhar et al. [54]. This shows the possibility of manipulating regions with varying number of graphene layers. This spatial selectivity of layers is desirable for engineering the properties for the device applications.

## Conclusions

In summary, the annealing effect of swift heavy ion irradiation on graphene in low-fluence regime has been observed. Raman studies show that 100-MeV Ag ion irradiation induced annealing up to a fluence of 3 × 10^11^ ions/cm^2^ after which a rapid increase in disorder is observed. The effect is similar to that shown earlier in fullerenes and CNTs. It is concluded that graphene can be purified by the use of SHI irradiation which is of particular importance for using CVD-grown graphene in device applications. Our results suggest that swift heavy ion with selected ranges of fluence may be used as one of the tools for defect annealing and manipulation of the number of graphene layers.

## Abbreviations

SHI: swift heavy ion; CNTs: carbon nanotubes; EBL: electron beam lithography; GFET: graphene FET; SEM: scanning electron microscope; MWCNT: multiwalled carbon nanotubes; CVD: chemical vapour deposition; pnA: particle nanoampere; AFM: atomic force microscope; FWHM: full width at half maxima; i-TS: inelastic thermal spike.

## Competing interests

The authors declare that they have no competing interests.

## Authors' contributions

SK carried out the experiments and data analysis and drafted the manuscript. AT and DKA contributed in the design and discussion of this work and in the revision of the manuscript, SAK carried out the theoretical simulations and fitting of curve. FS and VB carried out the Raman characterization and helped in the analysis of data. All authors read and approved the final manuscript.
